# U-shaped association between online information exchange and app usage frequency: a large-scale survey of China ‘s online young and middle-aged people with pre diabetes and diabetes

**DOI:** 10.3389/fendo.2023.1141645

**Published:** 2023-04-21

**Authors:** Hanbin Guo, Yibiao Xiao, Canlin Liao, Jiating Sun, Yanchun Xie, Yitong Zheng, Guanhua Fan

**Affiliations:** Shantou University Medical College, Shantou, China

**Keywords:** diabetes, online, app usage frequency, information, U-shape

## Abstract

**Background:**

China has the world’s largest diabetic population, and the cost of caring for all these people every day is substantial. Online information exchange and app usage frequency have been demonstrated to play a significant influence in the management of blood glucose and enhancement of diabetes-related quality of life. However, the association between online information exchange and app usage frequency among actual online populations remains unclear and deserves additional study. Therefore, we evaluated the factors affecting the frequency of app usage in the online glucose management population, with a particular emphasis on the connection between online information exchange and app use frequency, contributing to the expansion of the research of diabetes management models.

**Method:**

This cross-sectional study was conducted by disseminating questionnaires in blood glucose management-related forums and WeChat groups and included 1586 online users concerned about blood glucose management. Information exchange and app usage frequency were considered as independent and dependent variables, respectively. We performed stratified and single factor analysis, multiple equation regression analysis, smooth curve fitting, and threshold effect and saturation effect analysis. R (version 4.1.3, http://www.Rproject.org) and EmpowerStats were used for data analysis.

**Result:**

After adjusting for other covariates, information exchange was independently and positively associated with app use frequency (β = 8.6, 95% CI: 6.5 to 11.2, p < 0.001). Through interaction analysis, the most significant interaction factors influencing the relationship between information exchange and app usage frequency were identified as health insurance status, whether living with parents, glycated hemoglobin status in the previous month, and self-monitoring of blood glucose (SMBG). The association between information exchange and app usage frequency is U-shaped, with information exchange inflection points of 3.0 and 4.2. Information exchange and app usage frequency are negatively correlated when the average information exchange score is less than 3.0, and for every point increase in the average information exchange score, the likelihood of the app high usage frequency group compared to the app low usage frequency group decreases by 70%. The relationship between information exchange and app usage frequency is strongest when it is greater than or equal to 3.0 and less than or equal to 4.2. The probability of the app high usage frequency group occurring compared to the app low usage frequency group rises 17.3 times for every 1 point increase in the average information exchange score. The probability of the app high usage frequency group occurring in comparison to the app low usage frequency group increased by 1.8 times for every 1 point rise in information exchange when the average information exchange score was higher than 4.2.

**Conclusion:**

Age, body mass index, married, living with parents, hemoglobin level, SMBG, and information exchange were positively connected with app usage frequency in our study of online blood glucose management population. The link between information exchange and app use frequency was significantly U-shaped. The app usage frequency changed the most with the rise in information exchange when the information exchange score was greater than or equal to 3.0 and less than or equal to 4.2. Therefore, we ought to offer effort to concentrate on and increase the health-related behaviors and activities of those in this score interval.

## 1 Introduction

In 2019, the International Diabetes Federation estimated that 9.3% (463 million) people worldwide have diabetes, and that figure is expected to progressively increase, rising by 1.6% (337 million) by 2045. Despite this, approximately 50% of persons with diabetes are unaware of their condition (1). Long-term blood sugar instability will lead to diabetes-related complications (2), and put more of a strain on Medicare’s budget for diabetes treatment (3). Fortunately, online interventions found on the Internet play an encouraging role in lowering the risk of diabetes (4). Therefore, the development of simple and effective blood glucose interventions that make it possible for the general population to participate in them is the current focus of diabetes management in China (5–7).

Online information sharing has grown commonplace since the start of the internet era (8). Online information exchange consists mostly of information searching and information sharing. Users can seek information by browsing or posting questions, and they can give information by participating in or initiating forums or by submitting responses to inquiries (8, 9). As far as we know, online users are now also changing from one-way information searching to two-way information sharing (10). Users’ perceived social support, blood glucose management, self-care compliance, and quality of life are all enhanced through online information exchange (11, 12). As online information exchange enables a greater number of users to participate, it increases users’ pleasure and sense of belonging to online activities, which will lead to a favorable impression of online activities and encourage them to continue to engage in online activities (11, 13, 14).

In recent years, mobile health has increasingly been utilized as an innovative method for diabetes prevention and treatment (15). The number of people who utilize mobile health services is, as we all know, steadily increasing as a result of the popularity of these services and the ease with which they can be accessed (16). It has been proven that mobile health has a substantial effect on both glucose management and weight control (17, 18). In addition, mHealth contributes to an increase in diabetes awareness and medication adherence (19). Mobile health apps are widely used because of their many benefits, which include data storage, instruction, and assistance (20). Surprisingly, it has been demonstrated that information interchange through an app is superior to conventional diabetes management strategies for the self-management of people with diabetes (21, 22). The app’s wide audience means it can serve as a platform for cross-border communication and trade, unlocking new possibilities for mobile devices (23).

Results from mobile health interventions are significantly correlated with how often their target population engages in online activities (24), however, it is also influenced by socio-demographic variables such as age, gender, level of education, and health status (25). Furthermore, it is influenced by the app’s features, such as information acquisition and resource discovery (26). According to our knowledge, whether participants have health difficulties or the app has numerous virtual or actual incentives, they are more likely to use it (27). As the frequency of online engagement rises, the behavior of users in day-to-day management may shift (28), while more frequency app usage correlates with better health habits and more normal blood sugar levels (29, 30), without increasing the risk of hypoglycemia (30). Since the app usage frequency is influenced by numerous variables and swings over time (27), it is difficult to maintain a high frequency of use (25, 26). Therefore, it is important for us to find the factors that affect the frequency of app use.

App stickiness refers to the frequency and duration of app usage, and information exchange is a crucial factor in promoting app stickiness, with this link being especially prominent in social and email apps (31–33). According to Chin-Lung et al., social factors like information exchange will positively impact the persistence of app usage by acting directly on users’ motivations to keep using the app (32). However, investigations on the frequency of app use indicated that there was no statistically significant difference between the app management group’s 10.7 (SD = 9.5) and app information exchange management group’s 11.1 (SD = 7.3) app uses per week (p = 0.83) (34). Unfortunately, there are no research on the correlation between online information exchange and app usage frequency, and it is vital to investigate this correlation in order to acquire a deeper understanding of the mechanisms underlying blood glucose management.

Due to the significant relationship between the frequency of app use and daily blood glucose management (30), in order to effectively manage blood glucose, we need to improve the frequency of app use and create interventions to enhance the frequency of app use. According to recent research, apps with real-time information exchange capabilities can better follow online trends (31). According to research by Litchman et al., those who exchange information regularly are more invested in their online activities (35). Shao et al. observed that information exchange increases user satisfaction, which has an effect on the growth of social networks usage (33). However, research into the correlation between information exchange and app usage within digital communities is lacking. We hypothesize that information exchange is a driving force behind regular app use.

The objectives of this study were to determine the relationship between information exchange and app usage frequency, identify factors that affect app usage frequency, and predict daily online behavior of the online glucose management population, particularly app usage frequency, in order to help blood glucose management and find a new online behavioral pathway to effectively assist in blood glucose control.

## 2 Materials and methods

### 2.1 Participants

After accepting the researcher’s explanation of the purpose and significance of this study and instructions for completing the questionnaire, Chinese online blood glucose management users agreed to accept and complete the electronic questionnaire on their own or with the help of others to become study participants. Participants must meet the following inclusion criteria: First, participants with abnormal blood glucose (including diabetes: fasting blood glucose ≥7.0 mmol/L, 2-hour postprandial blood glucose ≥11.1 mmol/L; prediabetes: fasting blood glucose 6.1-6.9 mmol/L, 2-hour postprandial blood glucose <7.8 mmol/L; impaired fasting blood glucose: fasting blood glucose 5.6-6.1 mmol/L, 2-hour postprandial blood glucose <7.8 mmol/L; abnormal glucose tolerance: fasting blood glucose <7.0mmol/L, 2 hours postprandial blood glucose 7.8-10.9mmol/L), and had been exchanging information online and using the blood glucose management app. Second, participants with normal blood glucose who have interacted with people with abnormal blood glucose, participated in online information exchange activities, and use a blood glucose management app, and who wish to receive assistance and support in this area. Third, participants capable of completing the questionnaire on their own or with the aid of others. Fourth, there was no history of mental illness or cognitive impairment among the participants.

### 2.2 Data collection

Based on previously conducted studies, we created a thorough questionnaire for this cross-sectional study that included pertinent inquiries about information exchange and app usage frequency (36–38). We evaluated the features of online behavior and the elements that influence app usage frequency and analyzed data on online blood glucose management population characteristics, information exchange behavior, and app usage frequency. We demonstrated reliability through preliminary trials.

In this study, the individuals responsible for administering the questionnaires received professional training. Patients with diabetes who met the inclusion criteria were sent electronic questionnaires *via* the platform “Questionnaire Star” from March 15 to May 15, 2022. These questionnaires were posted in blood glucose management posting bars (such as the Sweet Home Bar, Blood Sugar Bar, High Blood Sugar Bar, Diabetes Bar, etc.), QQ groups (Diabetes Exchange Group), and blood glucose management WeChat groups (Glucose Friends Support Exchange Group). Based on the following exclusion criteria: (1) any two or more questionnaires with the same network IP address; (2) questionnaires with highly repetitive answers; (3) questionnaires that took less than 120s to complete; (4) questionnaires with illogical or contradictory content or omissions in the responses, 336 questionnaires were excluded and 1586 questionnaires were ultimately included. 82.9% of tests were valid.

**Figure 1:**
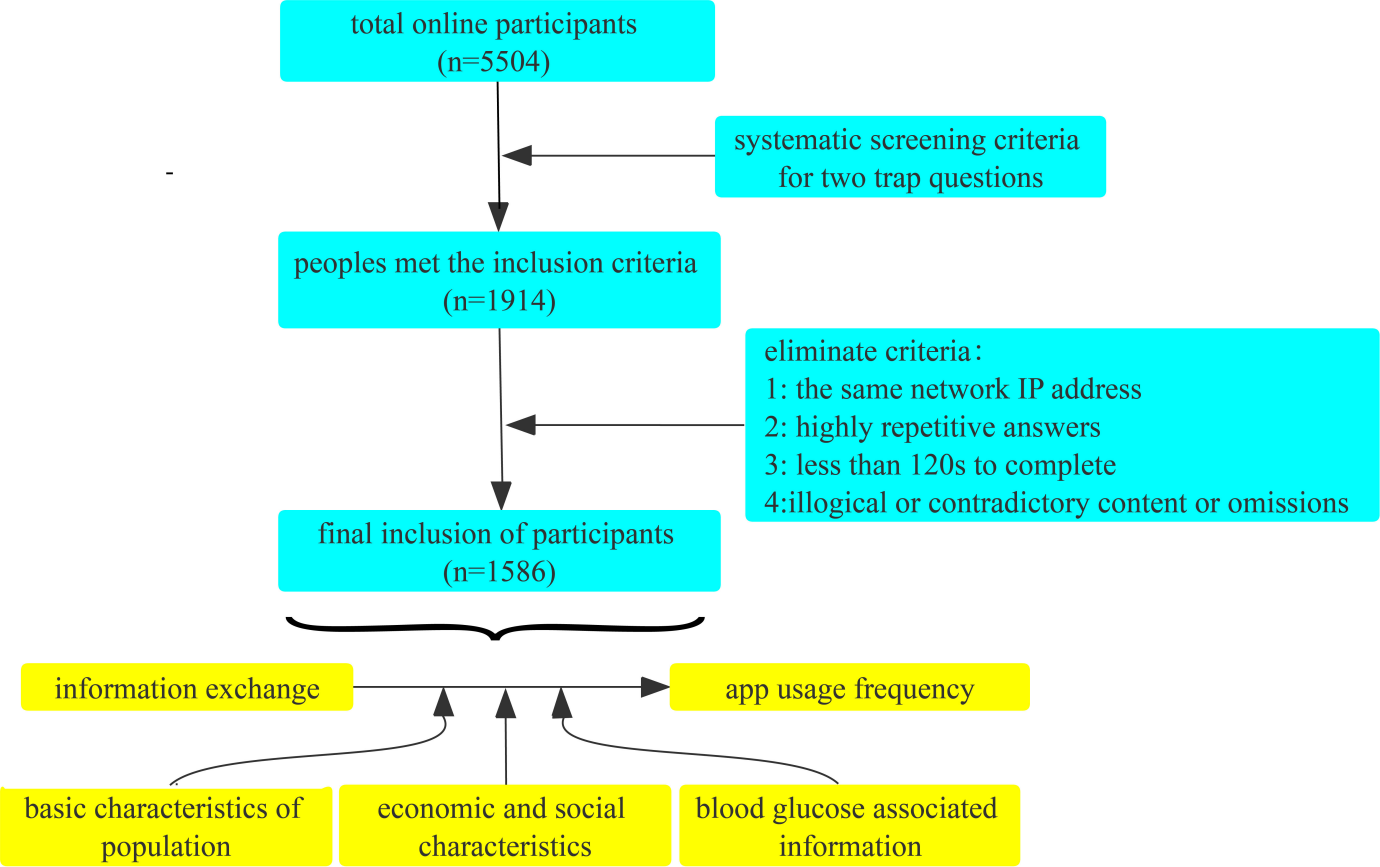
Research flow chart.

The questionnaire included demographic data (age, gender, height, weight, education, residence, marital status), social support status (family size, whether living with parents), blood glucose related information (glycosylated hemoglobin status, SMBG), active information searching frequency (before joining the online group, after joining the online group), online information exchange scale (5 items; valid and reliable, Cronbach’s α= 0.85>0.80 and KMO= 0.85>0.800, respectively) and app usage frequency scale (5 items; valid and reliable, Cronbach’s α= 0.79>0.70 and KMO= 0.79>0.70, respectively), The assessment of the frequency of active searching for online health-related information was based on two items on a 5-point Likert scale: 1 = “never” (0 searches per day), 2 = “occasionally” (1-2 searches per day), 3 = “generally” (3-5 searches per day), 4 = “sometimes” (6-10 searches per day), and 5 = “often” (more than 10 searches per day) with the following content: (1) before joining the online group; (2) after joining the online group. Online information exchange was assessed based on a 5-point Likert scale (1 = “strongly disagree”, 2 = “disagree”, 3 = “neutral”, 4 = “agree”, and 5 = “strongly agree”) of 5 items, which are as follows: (1) The exchange of knowledge and experience related to the group or forum is very convincing to me; (2) The members of the group or forum exchange knowledge and experience frequently, seriously, and enthusiastically; (3) I can ask relevant questions and answer them for more informative answers to my inquiries; (4) I can exchange more symptoms, conditions, or ideas with members of the group or forum; (5) When I feel confused or uncomfortable, members of the group or forum will provide me with useful online help or information. The evaluation of app usage frequency was based on 5 items on a 6-point Likert scale (1 = “never”, 2 = “less than once a month”, 3 = “once a month”, 4 = “several times a month”, 5 = “several times a week”, 6 = “every day”) with the following content: (1) WeChat (including WeChat groups), (2) Weibo, (3) QQ (including QQ groups), (4) Jitterbug (5) others. The above-mentioned scales with good reliability were used for patient self-assessment. The scores of the scales were determined by calculating the mean of the scale items. The flow chart of the study is shown in **Figure 1**.

### 2.3 Ethics approval and consent to participate

All participants provided verbal informed consent to inclusion before they participated in the study, and the protocol was approved by the Ethics Committee of the Medical College of Shantou University (Code: SUMC-2021–064).

### 2.4 Statistical analysis

Continuous variables are expressed as mean ± standard deviation and for categorical variables as number of cases (N, N%), ratio (OR) and 95% confidence interval (CI), respectively. For app usage frequency, we first convert it into a dichotomous variable based on the median, with less than 50% being the app low usage frequency group and greater than or equal to 50% being the app high usage frequency group. Information exchange was transformed into a trichotomous variable according to the trichotomous method, where the low information exchange group scored ≤ 3.8, the medium information exchange group scored > 3.8 and ≤ 4.2, and the high information exchange group scored > 4.2. Using single-factor and multi-factor binary logistic regression models, in order to further analyze the relationship between online information exchange and app usage frequency, we used three different models, model 1: no adjustment for variables; model 2: adjustment for sex and age; model 3: adjustment for sex, age, BMI, education, medical insurance, marital status, residence, family size, whether living with parents, glycated hemoglobin, and SMBG. In the sensitivity analysis, we also use the trichotomous variables of information exchange obtained by the trichotomous method, and calculate the trend to observe the possibility of nonlinearity. A weighted generalized additive model and a smooth curve fitting were performed to address nonlinearity between information exchange and app usage frequency. When nonlinearity was identified, the critical inflection point was determined using a recursive method. Then, a two-piecewise linear regression model was conducted on both sides of the inflection point. Interaction tests were conducted to assess whether patient characteristics influenced the association between online information exchange and app usage frequency. In addition to further study of the data, we subsequently performed subgroup analyses. The statistical analyses were conducted using package R (version 4.1.3, http://www.Rproject.org) and EmpowerStats software (http://www.empowerstats.com).

## 3 Results

### 3.1 Descriptive statistical analysis of participants

**Figure 2:**
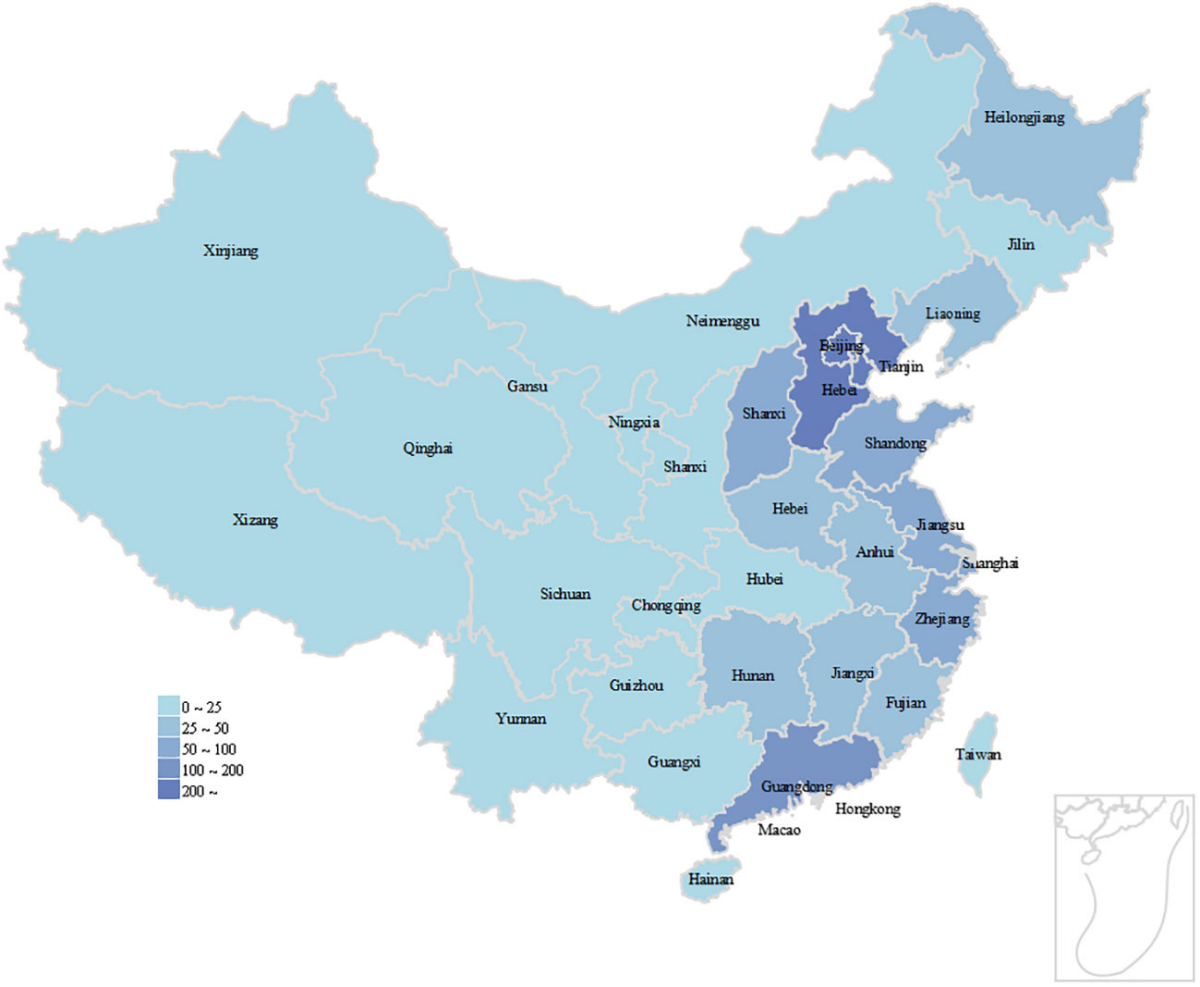
Distribution of participants’ region of residence.

**Figure 3:**
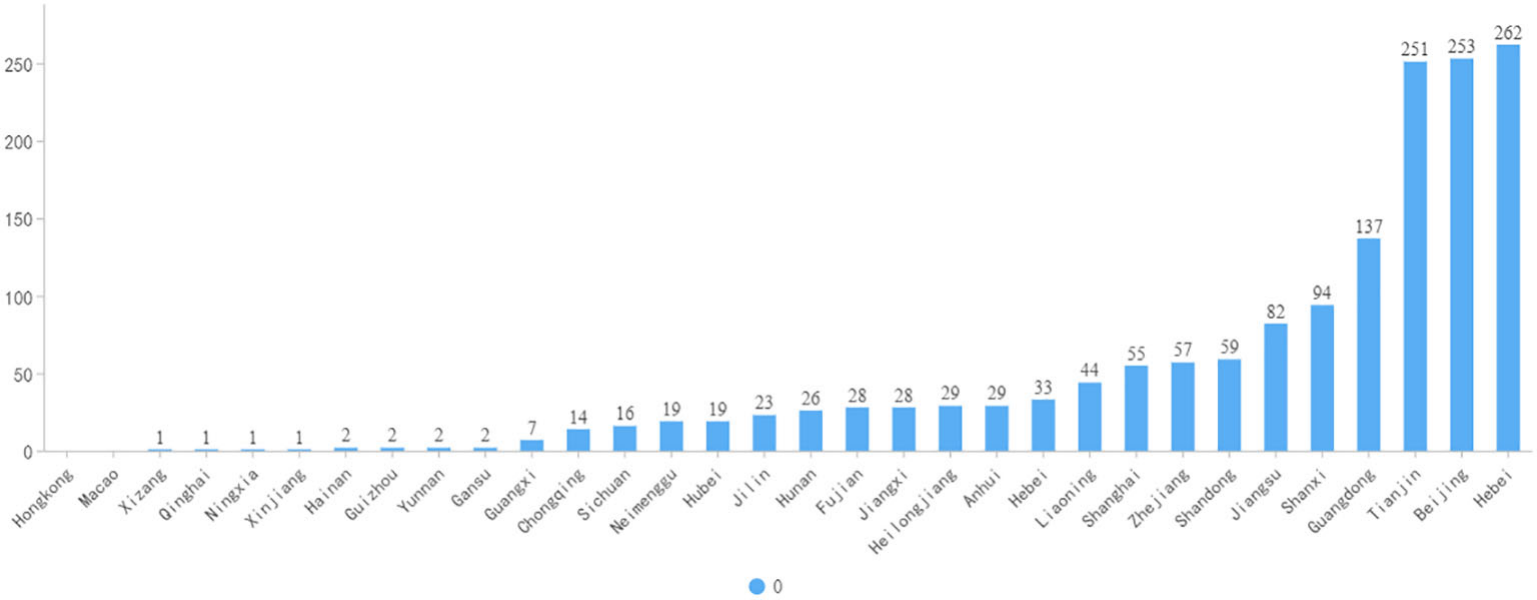
Participants’ provinces of residence.

Participants’ ages ranged from 29 to 90, with a median of 54 years old, and there were 1,586 blood glucose managers analyzed in this study. Among the entire population, 1,321 individuals (83.3% of the total) were <40 years old, whereas 265 individuals (16.7% of the total) were ≥40 years old. Of the total number of participants, 679 were female (42.8%) and 907 were male (57.2%). More than half of the participants have a normal BMI, and there are 1358 participants with a BMI between 18.5 and 24 who make up 66.90% of the total. The average score for information exchange was 4.1 ± 0.6. There were no statistically significant differences in the proportions of gender, education, BMI, medical insurance, residence, or family size between groups with high and low app usage frequency. In contrast, there were statistical differences in age, marital status, whether living with parents, glycosylated hemoglobin, SMBG and information exchange scores between groups with high and low app usage frequency (**Table 1**). From the IP address of the respondents, the respondents came from 32 provinces, autonomous regions and municipalities directly under the Central Government of China, of which Hebei, Beijing and Tianjin ranked the top three, which can be seen in **Figure 2**. Due to the wide geographical distribution of participants, there are many participants in each region, so the results of this study are representative of all regions in China. **Figure 3** maps the number of respondents in each region.

**Table 1:** Baseline characteristics of eligible participants, including their online app usage frequency classifications.

App usage frequency	Low	High	Total	P-value
N	673	913	1586	
Information exchange	3.8 ± 0.6	4.3 ± 0.4	4.1 ± 0.6	<0.001
Sex				0.928
male	384 (57.1%)	523 (57.3%)	907 (57.2%)	
female	289 (42.9%)	390 (42.7%)	679 (42.8%)	
Age				0.017
<40	578 (85.9%)	743 (81.4%)	1321 (83.3%)	
≥40	95 (14.1%)	170 (18.6%)	265 (16.7%)	
BMI				0.706
<18.5	86 (13.4%)	102 (11.4%)	188 (12.2%)	
>=18.5, <24	430 (66.9%)	609 (68.1%)	1039 (67.6%)	
>=24, <28	108 (16.8%)	157 (17.6%)	265 (17.2%)	
>=28	19 (3.0%)	26 (2.9%)	45 (2.9%)	
Education				0.247
junior high school and below	36 (5.3%)	36 (3.9%)	72 (4.5%)	
high school/technical secondary school/technical school	105 (15.6%)	163 (17.9%)	268 (16.9%)	
junior college	171 (25.4%)	200 (21.9%)	371 (23.4%)	
undergraduate	325 (48.3%)	465 (50.9%)	790 (49.8%)	
postgraduate and above	36 (5.3%)	49 (5.4%)	85 (5.4%)	
Medical security				0.826
urban employees’ medical insurance	242 (36.0%)	330 (36.1%)	572 (36.1%)	
urban residents’ medical insurance	179 (26.6%)	226 (24.8%)	405 (25.5%)	
new rural cooperative medical insurance	169 (25.1%)	235 (25.7%)	404 (25.5%)	
others	83 (12.3%)	122 (13.4%)	205 (12.9%)	
Marital status				<0.001
single or unmarried	238 (35.4%)	228 (25.0%)	466 (29.4%)	
married	416 (61.8%)	667 (73.1%)	1083 (68.3%)	
divorced or widowed	19 (2.8%)	18 (2.0%)	37 (2.3%)	
Residence				0.176
Urban	391 (58.1%)	559 (61.2%)	950 (59.9%)	
Suburban	154 (22.9%)	215 (23.5%)	369 (23.3%)	
Township	95 (14.1%)	110 (12.0%)	205 (12.9%)	
Countryside	33 (4.9%)	29 (3.2%)	62 (3.9%)	
Family size				0.092
≤3 persons	348 (51.7%)	511 (56.0%)	859 (54.2%)	
≥4 persons	325 (48.3%)	402 (44.0%)	727 (45.8%)	
Cohabitation with parents				<0.001
Yes	511 (75.9%)	761 (83.4%)	1272 (80.2%)	
No	162 (24.1%)	152 (16.6%)	314 (19.8%)	
Glycosylated hemoglobin				<0.001
≤6.4%	373 (55.4%)	529 (57.9%)	902 (56.9%)	
≥6.5%	203 (30.2%)	329 (36.0%)	532 (33.5%)	
Not tested	97 (14.4%)	55 (6.0%)	152 (9.6%)	
SMBG				<0.001
Not tested or occasionally tested	500 (74.3%)	516 (56.5%)	1016 (64.1%)	
Daily testing	173 (25.7%)	397 (43.5%)	570 (35.9%)	
Information exchange tertile				<0.001
Low	373 (55.4%)	150 (16.4%)	523 (33.0%)	
Medium	167 (24.8%)	259 (28.4%)	426 (26.9%)	
High	133 (19.8%)	504 (55.2%)	637 (40.2%)	

Divide into two groups according to the median of app usage frequency, less than 50% is the low usage frequency group of app and more than or equal to 50% is an app high usage frequency group; Information exchange was transformed into a trichotomous variable according to the trichotomous method, where the low information exchange group scored ≤ 3.8, the medium information exchange group scored > 3.8 and ≤ 4.2, and the high information exchange group scored > 4.2.

**Figure 4:**
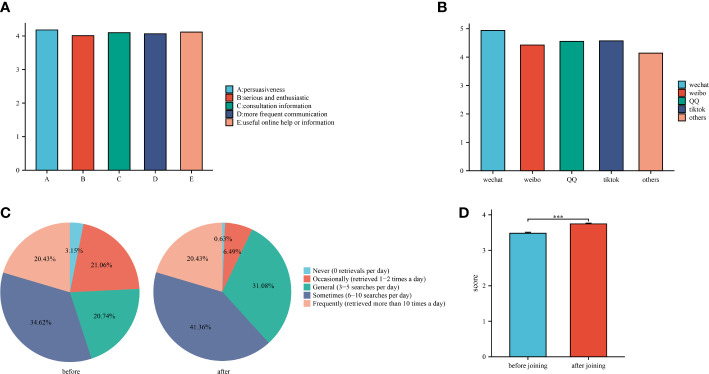
**(A)**: Scores for different online information exchange behavior; **(B)**: Score for different app usage frequency. **(C)**: Proportion of active search frequency before and after joining an online group. **(D)**: Average score of active search frequency before and after joining an online group.

**Figure 4A** demonstrates while the information exchange has the best score for information credibility (4.12 points), while it has the lowest score for user sincerity and passion (4.01 points). Overall, participants rated the online information exchange highly on average. WeChat has the greatest usage frequency score, accounting for 4.94 points in **Figure 4B**, followed by Tiktok, QQ, and Weibo. Other app is utilized less frequently than the above four online information exchange applications, with only 4.14 points. **Figures 4C, D** show that most people used active search for online health-related information both before and after joining the online group. Prior to joining the online group, participants scored 3.481 (SD=1.127) for online search, while after joining the online group, they scored 3.745 (SD=0.876), an increase in score compared to before joining that was statistically significant (p<0.001).

### 3.2 The relationship between online information exchange and app usage frequency

App use frequency increased significantly with information exchange score (p<0.05). The frequency of application use was higher among participants who were at least 40 years old, married, cohabited with their parents, had an elevated hemoglobin level in the previous month, and had regular daily blood glucose testing(p<0.05). However, the frequency of app use was not affected by gender, BMI, education, medical insurance, residence, or family size (**Table 2**).

**Table 2:** Univariate analysis for app usage frequency.

	Statistics	App usage frequency
Sex		
male	907 (57.2%)	1.0
female	679 (42.8%)	1.0 (0.8, 1.2) 0.928
Age		
<40	1321 (83.3%)	1.0
≥40	265 (16.7%)	1.4 (1.1, 1.8) 0.018
BMI		
<18.5	188 (12.2%)	1.0
>=18.5, <24	1039 (67.6%)	1.2 (0.9, 1.6) 0.266
>=24, <28	265 (17.2%)	1.2 (0.8, 1.8) 0.291
>=28	45 (2.9%)	1.2 (0.6, 2.2) 0.670
Education		
junior high school and below	72 (4.5%)	1.0
high school/technical secondary school/technical school	268 (16.9%)	1.6 (0.9, 2.6) 0.099
junior college	371 (23.4%)	1.2 (0.7, 1.9) 0.543
undergraduate	790 (49.8%)	1.4 (0.9, 2.3) 0.146
postgraduate and above	85 (5.4%)	1.4 (0.7, 2.6) 0.338
Medical insurance		
urban employees’ medical insurance	572 (36.1%)	1.0
urban residents’ medical insurance	405 (25.5%)	0.9 (0.7, 1.2) 0.557
new rural cooperative medical insurance	404 (25.5%)	1.0 (0.8, 1.3) 0.882
others	205 (12.9%)	1.1 (0.8, 1.5) 0.650
Marital status		
single or unmarried	466 (29.4%)	1.0
married	1083 (68.3%)	1.7 (1.3, 2.1) <0.001
divorced or widowed	37 (2.3%)	1.0 (0.5, 1.9) 0.974
Residence		
Urban	950 (59.9%)	1.0
Suburban	369 (23.3%)	1.0 (0.8, 1.2) 0.849
Township	205 (12.9%)	0.8 (0.6, 1.1) 0.173
Countryside	62 (3.9%)	0.6 (0.4, 1.0) 0.064
Family size		
≤3 persons	859 (54.2%)	1.0
≥4 persons	727 (45.8%)	0.8 (0.7, 1.0) 0.092
Cohabitation with parents		
yes	1272 (80.2%)	1.0
no	314 (19.8%)	0.6 (0.5, 0.8) <0.001
Glycosylated hemoglobin		
Not tested	152 (9.6%)	1.0
≤6.4%	902 (56.9%)	2.5 (1.8, 3.6) <0.001
≥6.5%	532 (33.5%)	2.9 (2.0, 4.2) <0.001
SMBG		
Not tested or occasionally tested	1016 (64.1%)	1.0
Daily testing	570 (35.9%)	2.2 (1.8, 2.8) <0.001
**Information exchange**	4.1 ± 0.6	8.2 (6.4, 10.5) <0.001
Information exchange tertile		
Low	523 (33.0%)	1.0
Medium	426 (26.9%)	3.9 (2.9, 5.1) <0.001
High	637 (40.2%)	9.4 (7.2, 12.3) <0.001

Divide into two groups according to the median of app usage frequency, less than 50% is the low usage frequency group of app and more than or equal to 50% is an app high usage frequency group; Information exchange was transformed into a trichotomous variable according to the trichotomous method, where the low information exchange group scored ≤ 3.8, the medium information exchange group scored > 3.8 and ≤ 4.2, and the high information exchange group scored > 4.2.

In this study, we developed three models to determine if information exchange has an independent effect on app usage frequency. Adjusted and unadjusted data are used to examine the relationship between information exchange and app usage frequency in **Table 3**. Using the information exchange score as a continuous and categorical variable, the relationship between information exchange and app usage frequency was confirmed. Model 1 and 2 found a positive correlation between information exchange and app usage frequency when information exchange was modeled as a continuous variable (OR=8.2, 95% CI: 6.4 to 10.5, p<0.001; OR=8.3, 95% CI: 6.4 to 10.6, p<0.001, respectively). In model 3, we also found a similar correlation (OR=8.6, 95% CI: 6.5 to 11.2, p<0.001). Using the trichotomous approach, information exchange was positively associated with app usage frequency in Model 1 (OR = 9.4, 95% CI: 7.2 to 12.3, p<0.001). Similarly, we found no significant difference between model 2 and 3 compared with model 1 (OR=9.5, 95% CI: 7.2 to 12.5, p<0.001; OR=9.6, 95% CI: 7.2 to 12.9, p<0.001 respectively). In the sensitivity analysis, we also calculated p-values for trends to observe the likelihood of non-linearity based on information exchange trichotomous variables derived from the trichotomous method, and we observed comparable trends (p<0.01).

**Table 3:** Multiple regression equation analysis for the relationship between online information exchange and app usage frequency.

Outcome	Model 1	Model 2	Model 3
	β (95%CI) P-value	β (95%CI) P-value	β (95%CI) P-value
Information exchange	8.2 (6.4, 10.5) <0.001	8.3 (6.4, 10.6) <0.001	8.6 (6.5, 11.2) <0.001
Information exchange tertiles			
Low	1.0	1.0	1.0
Medium	3.9 (2.9, 5.1) <0.001	3.9 (3.0, 5.1) <0.001	4.0 (2.9, 5.3) <0.001
High	9.4 (7.2, 12.3) <0.001	9.5 (7.2, 12.5) <0.001	9.6 (7.2, 12.9) <0.001
Information exchange tertile trend	3.1 (2.7, 3.5) <0.001	3.1 (2.7, 3.5) <0.001	3.1 (2.7, 3.6) <0.001

Non-adjusted model adjust for: None.

Adjust I model adjust for: sex; age.

Adjust II model adjust for: sex; age; BMI; education; medical insurance; marital status; residence; family size; cohabitation with parents; glycosylated hemoglobin; SMBG.

**Figure 5:**
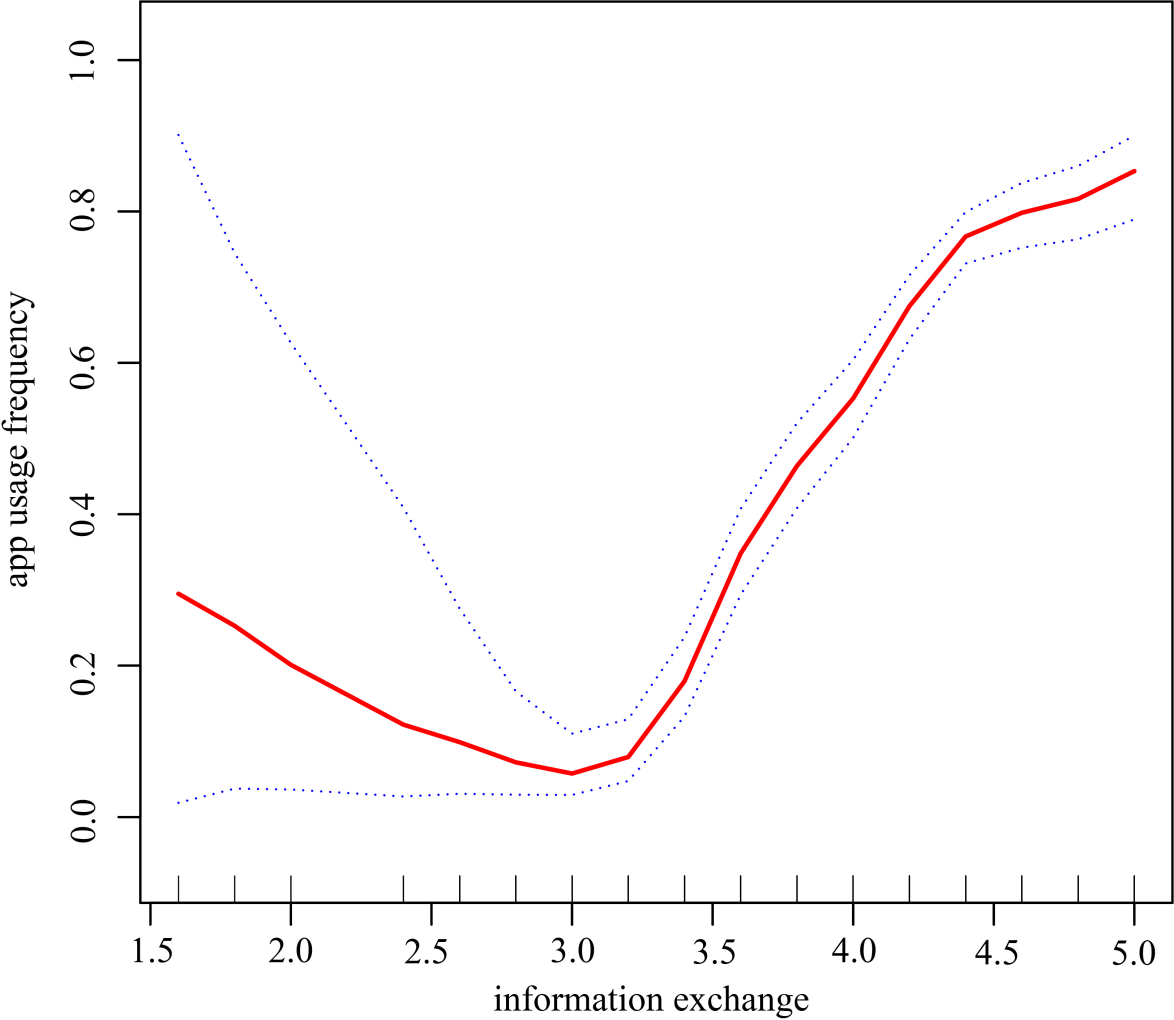
Analysis of the relationship between information exchange and app usage frequency in the online blood glucose management population by smoothing curve fitting The red solid bars indicate a smooth curve fit between information exchange and app usage frequency in the blood glucose management population, and the blue dashed strip indicates the 95% confidence interval of the fit between information exchange and app usage frequency. Adjusting variables: sex; age; BMI; education; medical insurance; marital status; residence; family size; cohabitation with parents; glycosylated hemoglobin; SMBG Note:Divide into two groups according to the median of app usage frequency, less than 50% is the low usage frequency group of app and more than or equal to 50% is an app high usage frequency group.

As information exchange is a continuous variable, it is necessary to examine the nonlinear connection between online information exchange and app usage frequency. Using a generalized additive model and a smoothed curve fit, we then sought to establish a connection between online information exchange and app usage frequency. The relationship between information exchange and app use frequency was nonlinear, as shown in **Figure 5**. (after adjusting for sex; age; BMI; education; medical insurance; marital status; residence; family size; cohabitation with parents; glycosylated hemoglobin and SMBG), and a descending and ascending curve can be seen from left to right. In **Table 4**, we employ a two-segment regression model in which the inflection points are 3.0 and 4.2. When the information exchange score was below 3.0, there was a negative correlation between information exchange and app usage frequency, with each 1-unit increase in information exchange reducing the probability of occurrence by 70% in the high app usage frequency group compared to the low app usage frequency group, but without a statistically significant difference in outcome (OR=0.3, 95% CI:0.1 to 1.7, p=0.169). When the information exchange score was between 3.0 and 4.2, there was a positive correlation between information exchange and app usage frequency, with a 17.3-fold increase in the probability of occurrence of the high app usage group for every 1-unit increase in the information exchange score (OR=18.3, 95% CI:11.0 to 30.4, p<0.001). For every 1 unit increase in the information exchange score, the app high usage frequency group increased by 1.8 times relative to the app low usage frequency group when the information exchange score was higher than 4.2 (OR=2.8, 95% CI:1.1 to 6.9, p<0.05).

**Table 4:** Independent correlation between online information exchange and app usage frequency analysis by multiple segmented linear regression.

Outcome:	App usage frequency		
	β	(95%CI)	P-value
Model I			
A straight-line effect	8.6	(6.5, 11.2)	<0.001
Model II			
Fold points (K1, K2)	3, 4.2		
< K1 segment effect 1	0.3	(0.1, 1.7)	0.169
K1-K2 segment effect 2	18.3	(11.0, 30.4)	<0.001
>K2 segment effect 3	2.8	(1.1, 6.9)	0.028
Log likelihood ratio tests	<0.001		

Adjusting variables: sex; age; BMI; education; medical insurance; marital status; residence; family size; cohabitation with parents; glycosylated hemoglobin; SMBG.

### 3.3 A subgroup analysis of the relationship between online information exchange and app usage frequency

Subgroup analyses were performed to investigate the connection between information exchange and app usage frequency. Besides, we also evaluated were the interrelationships among the subgroup’s various strata. As shown in **Table 5**, there is a positive and statistically significant correlation between online information exchange and app usage frequency across all study subgroups. There were no statistically significant differences by gender (p=0.7329), age (p=0.1363), BMI (p=0.9791), education (p=0.5576), marital status (p=0.2765), residence (p=0.6802), or family size (p=0.8488) after the interaction. In contrast, interaction tests were significant in different strata of the subgroups of medical insurance(p=0.0190), cohabitation with parents (p=0.0048), glycosylated hemoglobin (p=0.0076) and SMBG (p=0.0269).

**Table 5:** **(A)**subgroup analysis of the correlation between online information exchange and app usage frequency.

	N	App usage frequency	P for interaction
Sex			0.7329
Male	878	8.2 (5.8, 11.6) <0.0001	
Female	659	9.0 (6.0, 13.7) <0.0001	
Age			0.1363
<40	1277	9.3 (7.0, 12.6) <0.0001	
≥40	260	5.5 (3.0, 10.2) <0.0001	
BMI			0.9791
<18.5	188	7.7 (3.9, 15.1) <0.0001	
>=18.5, <24	1039	8.5 (6.2, 11.8) <0.0001	
>=24, <28	265	9.3 (4.8, 17.8) <0.0001	
>=28	45	9.9 (2.1, 47.0) 0.0041	
Education			0.5576
Junior high school and below	67	6.5 (2.0, 20.9) 0.0017	
High school/technical secondary school/technical school	261	6.9 (3.9, 12.2) <0.0001	
Junior college	359	10.1 (5.8, 17.5) <0.0001	
Undergraduate	766	8.1 (5.5, 11.9) <0.0001	
Postgraduate and above	84	20.2 (5.4, 75.5) <0.0001	
Medical security			0.0190
Urban employees’ medical insurance	563	5.8 (3.8, 8.9) <0.0001	
Urban residents’ medical insurance	387	8.4 (5.1, 13.9) <0.0001	
New rural cooperative medical insurance	391	17.7 (9.8, 31.8) <0.0001	
Others	196	6.9 (3.4, 14.3) <0.0001	
Marital status			0.2765
Single or unmarried	448	9.1 (5.7, 14.6) <0.0001	
Married	1056	7.9 (5.7, 11.0) <0.0001	
Divorced or widowed	33	40.6 (3.8, 428.5) 0.0021	
Residence			0.6802
Urban	929	8.2 (5.8, 11.5) <0.0001	
Suburban	355	9.2 (5.3, 15.8) <0.0001	
Township	197	11.2 (5.4, 23.2) <0.0001	
Countryside	56	4.8 (1.5, 15.9) 0.0101	
Family size			0.8488
≤3 persons	829	8.4 (5.8, 12.0) <0.0001	
≥4 persons	708	8.8 (6.0, 13.0) <0.0001	
Cohabitation with parents			0.0048
Yes	1236	10.5 (7.7, 14.3) <0.0001	
No	301	4.2 (2.5, 7.2) <0.0001	
Glycosylated hemoglobin			0.0076
Not tested	145	4.1 (2.0, 8.3) 0.0001	
≤6.4%	877	7.3 (5.2, 10.3) <0.0001	
>6.5%	515	15.1 (9.1, 25.2) <0.0001	
SMBG			0.0269
Not tested or occasionally tested	984	7.0 (5.2, 9.6) <0.0001	
Daily testing	553	13.5 (8.1, 22.4) <0.0001	

## 4 Discussion

There is growing evidence that online information exchange and regular app use play an important role in blood glucose control and daily blood glucose management, and these factors have become the focus of online users for blood glucose management (30, 39–41). We can see a statistically significant difference in app usage frequency in accordance with the extent of online information exchange, and this is the first large-scale study to our knowledge to explore the connection between information exchange and app usage among Chinese internet users.

### 4.1 Basic demographic characteristics

Among the survey population of respondents to the various clinical stages of blood glucose management included in this study, the ratio of males to females is roughly 1:1, and the population tends to be younger, with the number of people aged 40 years accounting for approximately 83.3% of the total. In addition, of the total number of participants, 55.2% have some college education. Similar findings were found in a Chinese online health community survey (42), proving that our sample is a good representation of the whole.

The fact that young people and student groups make up the bulk of China’s internet users may be related to the fact that they lack the knowledge to support their health management and the confidence to refute dubious and untrue opinions, so they prefer to communicate with people who have had similar experiences *via*the internet and rely on online health information to aid them in managing their health (43).

We observe that married people and cohabitation with their parents make up a significant portion of the high app usage frequency group, accounting for 73.1% and 83.3% respectively of the total. Evidence suggests this may have its origins in the advantages of family behavioral therapy, in which members of the family can actively communicate and take responsibility for one another in learning about and applying the latest developments in blood glucose management *via*the use of apps (44, 45). Brew-Sam et al., on the other hand, suggest that parental encouragement does not always lead to more app usage. Users may turn to third parties, such as blood glucose management apps, for help when they encounter unhelpful family behaviors. On the other hand, the need for self-care apps may be reduced if families are very involved in their loved ones’ day-to-day management (44). Those in our study population who were married or still living at home with their parents were more likely to use the app, which may have something to do with the relative stability of these households and their members’ willingness to lend a helping hand without being intrusive.

We were surprised to find that SMBG, as well as having a test for glycated hemoglobin, was also positively correlated with app usage frequency. Previous research has shown that patients who are successful at sticking to SMBG have higher levels of health-related knowledge, self-efficacy, and education (46), while all these factors work in favor of encouraging self-management of blood glucose (47, 48).

### 4.2 High score in online information exchange

This study’s research of the online population reveals that the online population scored high on information exchange, with scores of 4 or above, with the highest ratings for information trustworthiness and usefulness being 4.18 and 4.11, respectively. Although there is a wealth of information available on various online information exchange platforms, its veracity varies widely, and misinformation and falsehoods that have not been verified are widely disseminated. This leaves users feeling overwhelmed when trying to decide which platform is best for them to access information and communicate with others (49). As a result, managers of online platforms should increase their oversight of online information exchange platforms and monitor misleading information in real time, and the general public should train itself to be more critical in its information selection and identification rather than blindly following the herd. Currently, some online platforms include not only specialist institutions, but also people in similar situations who assist one another. Meanwhile, credibility of the data is ensured by the platform’s ongoing efforts to improve its management and introduce a system for reporting false information (50).

The frequency of active searches by online users increased after joining online groups relative to before joining online groups (before joining: 3.481 ± 1.127 *vs*after joining: 3.745 ± 0.876, p<0.001), which is because, once a user joins a group, they are more likely to be affected by the group’s members, to become more interested in health information, or to experience a decline in their own health (51). However, a cross-sectional study conducted by Kalantzi et al. at the Athens Diabetes Clinic found that users actively sought information in the beginning, but less so as time progressed and basic knowledge was learned (52). This contradicts our findings, possibly because our participants included may have been diagnosed with abnormal blood glucose or aware of blood glucose related issues for a relatively short period of time, and the participants were still in the knowledge building phase of a continuous active search. At present, the most frequently searched questions online remain relate to the pathophysiology, complications, diet, and prevention of diabetes, and the most reliable internet resources continue to come from medical specialists (52, 53). In order to create mobile health apps that help users better regulate their blood glucose levels in accordance with clinical standards, healthcare professionals and app developers should work together to better understand user preferences.

### 4.3 The relationship between information exchange and app usage frequency

Presently, China’s blood glucose management app primarily provides information on disease treatment, health status, self-care, psychosocial, laboratory test results, and information released by healthcare professionals. Online information learning and exchange among blood glucose management users has become an important method of blood glucose management (54). This online exchange is also gradually shifting from a purely receptive to a two-way exchange of information, which enables patients to gain emotional and social support from interacting with users who share their experiences, leading them to derive more benefit from, and become more enthusiastic about, participating in online activities (34). According to a study conducted by Jin et al., health information shared by doctors or other self-glucose managers in China’s largest online health community had an initiating and spreading effect, leading to the sugar users receiving the information becoming the dominant ones sharing the information and integrating themselves into the online internet (11). Increasing adherence to blood glucose management is aided by both faith in the Internet and the information exchange online, according to the results of another Chinese online poll (55). Previous research has found a link between health literacy and effective blood glucose self-management (11, 56). In a randomized controlled trial of diabetes patients lasting 6 months, Zhang et al. found a statistically significant drop in HbA1c of 2.03% in the app-interactive management group compared to 1.37% in the app-managed group, but no statistically significant difference in blood glucose self-monitoring (57), which means that information exchange is a potential factor in improving glycemic status and is a starting point for future research into glycemic control.

The blood glucose self-management potential of mobile health apps is substantial. In spite of the fact that mobile health apps simplify blood glucose self-management, not all users are successful in maintaining their routines. Therefore, it is particularly important to examine the factors that influence the frequency of app use. A meta-analysis that included 99 articles suggested that users are more likely to regularly check their mobile health app if it is tailored to their specific needs, provides alerts in the form of personalized push messages, is easy to navigate and operates smoothly technically, and offers personal support in addition to digital interventions (58). Other research indicate that online information exchange is a significant factor of app usage frequency, with this association being especially prominent in some social applications and email apps (31–33). It is worth emphasizing that information with visual characteristics boosts the user’s reliance on the app, which means that apps with graphic and video-based information have an edge in enticing people to use them (59). This is in line with our findings, with picture or video-based WeChat, Tiktok and QQ ranking in the top three for frequency of use, with scores of 4.97, 4.57 and 4.56 respectively. In Chin-Lung et al., it is suggested that social impacts, such as information exchange, have a beneficial effect on the frequency of app use (32). However, a different perspective has been proposed. A 6-month single-center prospective randomized controlled trial in China found that people who used the app for blood glucose self-management (with Welltang installed for self-management) used it 10.7 ± 9.5 times per week, while those who used the app for interactive management (with Welltang installed for self-management + information exchange interaction) used it 11.1 ± 7.3 times per week, and the difference was not statistically significant (p=0.83) (34). Unfortunately, there are no research on the association between online information exchange and app usage frequency, and it is necessary to analyze and quantify this relationship in order to obtain additional insight into the mechanisms of blood glucose management.

In our study, online information exchange scores were correlated with app use frequency across subgroups of age, gender, BMI, education, medical insurance, marital status, residence, family size, cohabitation with parents, glycated hemoglobin, and SMBG. The interaction test was significant (p<0.05) in the different strata of the subgroups with medical insurance derived from the interactivity calibration, with the online information exchange of the urban employees’ medical insurance population contributing least to app use frequency at 5.8 (3.8, 8.9) and the highest contribution among the new rural cooperative medical insurance population at 17.7 (9.8, 31.8). This is partly related to the low accessibility of health services in rural areas and the relative lack of medical knowledge (60, 61). Most of those who participate in the new rural cooperative medical care are from relatively backward rural areas, and their limited medical level and lack of medical knowledge lead them to look for ways to help with health management through the Internet, so when they engage in online information exchange, they are more motivated to learn how to use the Internet, so those who participate in the new rural cooperative medical care show a stronger promotion of app usage frequency by those who participate in the new rural cooperative medical care. An interaction test revealed a statistically significant difference (p<0.05) in the effect of online information exchange on the frequency of app use between those who lived with their parents and those who did not, which is directly related to the beneficial effect of family support in diabetes treatment. Family support can help users manage their health by reducing stress, changing their emotional states, enhancing self-efficacy, and encouraging negative health behavior modification, which is consistent with the increased frequency of app usage among individuals cohabiting with parents in our study (62).

Our research is the first to find a statistically significant U-shaped correlation between information exchange and app usage frequency among Chinese internet users and this correlation was unaffected by other potential dangers. The relationship between information exchange and app usage frequency is strongest when the average information exchange score is in the range of 3.0-4.2, with each increase in the average information exchange score increasing the high app usage frequency by 18.3 times, while when the average information exchange score is >4.2, each increase in the average information exchange score increases the high app usage frequency by 2.8 times. Once patients have a sense of belonging in the process of exchanging information, their online satisfaction rises, they begin to trust the app, and they are more likely to utilize it (63). However, we have also found that as users move from satisfied to very satisfied with the act of information exchange, the frequency of app use slows down, which may have to do with the fact that the more information is exchanged, the more users are able to identify mismatches between the app and their needs, and thus the app takes up less and less time in their lives (64). This may also explain why the more information people exchange, the less often they utilize apps.

### 4.4 Limitations

Although this is the first study to investigate the relationship between information exchange and app usage frequency, it does have some limitations. At first, our cross-sectional design precludes us from drawing any firm conclusions about the cause and effect of information exchange and app usage frequency; Secondly, some confounding factors that were not included may have had an impact on this study. Third, we have not yet validated the potential processes of information exchange and app usage frequency, which will be the focus of future research.

## 5 Conclusions

This study showed that among the online blood glucose management population, participants’ age, BMI, marital status, cohabitation with parents, glycosylated hemoglobin, SMBG, and information exchange scores were positively associated with app usage frequency, with online information exchange being independently associated with app usage frequency. We found a curvilinear relationship between online information exchange and app usage frequency. Online information exchange appears to be a risk factor for high app usage when the online information exchange score is <3.0, but can contribute to an increase in app usage when the information exchange score is ≥3.0, and this increase decreases when the score is >4.2. Therefore, online information exchange may be a simple and accurate way of predicting the frequency of app use for the online blood glucose management population.

## Data availability statement

The original contributions presented in the study are included in the article/supplementary material. Further inquiries can be directed to the corresponding author.

## Ethics statement

The studies involving human participants were reviewed and approved by The Ethics Committee of Shantou University Medical College. Written informed consent from the participants’ legal guardian/next of kin was not required to participate in this study in accordance with the national legislation and the institutional requirements.

## Author contributions

HG undertook the data analysis, complete statistical tables and result analysis, wrote the research process, literature reviews, and interpreted the results. YBX, CL, JS, YCX, YZ performed the design of the questionnaire and data collection, GF was in charge of the conception, undertook the design of the study framework, participated the data collection, complete the whole of statistical tables, takes responsibility for the integrity of the data and the accuracy of the data, revised the entire manuscript, and interpreted the conclusion. GF ultimately modified the manuscript. All authors contributed to the article and approved the submitted version.
